# Shifting gears: Diversification, intensification, and effort increases in small-scale fisheries (1950-2010)

**DOI:** 10.1371/journal.pone.0190232

**Published:** 2018-03-14

**Authors:** Jennifer C. Selgrath, Sarah E. Gergel, Amanda C. J. Vincent

**Affiliations:** 1 Project Seahorse, Institute for the Oceans and Fisheries, The University of British Columbia, Vancouver, British Columbia, Canada; 2 Forest and Conservation Sciences, The University of British Columbia, Vancouver, British Columbia, Canada; Aristotle University of Thessaloniki, GREECE

## Abstract

Locally sustainable resource extraction activities, at times, transform into ecologically detrimental enterprises. Understanding such transitions is a primary challenge for conservation and management of many ecosystems. In marine systems, over-exploitation of small-scale fisheries creates problems such as reduced biodiversity and lower catches. However, long-term documentation of how governance and associated changes in fishing gears may have contributed to such declines is often lacking. Using fisher interviews, we characterized fishing gear dynamics over 60 years (1950–2010) in a coral reef ecosystem in the Philippines subject to changing fishing regulations. In aggregate fishers greatly diversified their use of fishing gears. However, most individual fishers used one or two gears at a time (mean number of fishing gears < 2 in all years). Individual fishing effort (days per year) was fairly steady over the study period, but cumulative fishing effort by all fishers increased 240%. In particular, we document large increases in total effort by fishers using nets and diving. Other fishing gears experienced less pronounced changes in total effort over time. Fishing intensified through escalating use of non-selective, active, and destructive fishing gears. We also found that policies promoting higher production over sustainability influenced the use of fishing gears, with changes in gear use persisting decades after those same policies were stopped. Our quantitative evidence shows dynamic changes in fishing gear use over time and indicates that gears used in contemporary small-scale fisheries impact oceans more than those used in earlier decades.

## Introduction

Long-term assessment of natural resource exploitation is critical to understanding ecosystem change and how governance affects those changes [1]. Contemporary ecosystems are shaped by their history of disturbances and human interventions [2]. These historical impacts influence the distribution of species and habitats that we see today and can affect modern-day ecosystem functioning and services [3,4]. In addition, the historical context can inform managers and other actors tasked to understand current conditions and to direct future ecological change [5].

The need for a grounded historical perspective and the integration of non-traditional data sources are particularly apparent for small-scale fisheries [5–7]. Although small-scale fisheries use small boats with basic fishing equipment, they represent a critical component of global fisheries. Small-scale fisheries collectively employ over 230 million people in the direct and indirect sectors [8], and have edible catches rivaling those of industrial fisheries [9]. Widespread overfishing has led to international commitments to improve their sustainability (FAO Voluntary Guidelines for Securing Sustainable Small-Scale Fisheries) [10] and a call for research to assess the effectiveness of management approaches [11]. Although catch declines in these fisheries are often attributed to the shift towards unsustainable fishing activities, little long-term information exists to contextualize or quantify these changes [12]. In many cases, the trajectories of small-scale fisheries remain undocumented because they involve a large number of fishers (25 times more individuals than industrial fisheries), are decentralized, and occur in countries with limited governance, funds, and/or technical expertise [10]. In such data-poor systems, local environmental knowledge (LEK) can provide information about the history of ecosystems and the practices of humans who depend on them [7].

Evaluating the choice of fishing methods and associated gears—such as fish traps and drift-nets—deployed over time is one key to understanding small-scale fisheries [13]. Small-scale fisheries use many fishing gears and catch a plethora of fish and invertebrate species (e.g. cephalopods, gastropods). Fishers adapt gear usage for changing biophysical conditions (e.g. tides, depth), different habitats (e.g. coral, seagrass), as well as to incorporate species’ behavior [14]. Additionally, fishers can change gears and adjust the effort allocated to various gears in response to the availability of marine life, evolving biophysical conditions, and market competition [13–15]. Fishing intensification by the use of more efficient fishing gears and methods can provide higher catch efficiency and fisheries yields. Intensive fishing methods can be valuable for fishers and coastal communities when marine life populations are strained, but their higher catch efficiency may also come with greater environmental costs [16]. For example, some gears with high catch efficiency also damage marine habitats [17] or catch non-target and juvenile marine life (i.e. bycatch) [18]. Thus, trends in gear use can be used to identify trends in fishing intensification and infer fishing impacts to ecosystems.

The management and governance of small-scale fisheries can incorporate a variety of approaches, but often include gear restrictions and protected areas [19]. Management as well as institutional approaches to governance change over time, reflecting dynamic societal priorities and values [20]. For example, governance that prioritizes the goals of a central government frequently maintains hierarchical management institutions. In contrast, governance that values local empowerment may develop co-management institutions based on local participation [21]. In many countries, institutional priorities have swung from an emphasis on extraction and resource exploitation in the 1970s and 1980s [22] to a contemporary focus on sustainability [10,23]. The extent to which resource users change their practices in step with governance priorities is influenced by how strongly past practices constrain and guide future choices (i.e. the “memory” of the system) [24].

The need for effective management is particularly relevant in small-scale fisheries targeting coral reefs in the Philippines, a country of global priority for biodiversity conservation [25]. Coral reefs are among the most productive and biologically diverse ecosystems in the world, but face many stressors including climate change, destructive fishing, overfishing, and pollution [26,27]. At the apex of the Coral Triangle, the Philippines is located in the global epicenter of marine biodiversity [28] and contains the third most extensive reef system in the world (about 22,000 km^2^) [29]. The country is highly dependent on fishing for food and livelihoods [30]. Reef fisheries in the Philippines once provided up to 37 tons per km^2^ per year, but catches throughout the country have sharply declined [31–33]. Due to its combination of high biodiversity and high threats, the management of Philippine small-scale fisheries is an important challenge globally.

In this study, we characterize 60 years of fishing gear dynamics in a small-scale fishery in the central Philippines. We examine a time period (1950–2010) coinciding with extensive changes in fisheries governance, ecosystem degradation, declining catches, and increasing populace [34–37]. We ask two questions. First, do historical trends in the use of fishing gears indicate intensification and/or a growing ecological impact from these fisheries? Second, is there evidence that fishing gear trends are influenced by changes in fisheries governance priorities? As no long-term records of fishing practices exist in our study area, we used local environmental knowledge to document the history of fishing gear use, including changes in the diversity of gears in use and the use of illegal gears. We also evaluated changes in fishing intensity as reflected by fishing effort and the use of active, non-selective, and destructive gears.

## Methods

### Study site

**Fig 1:**
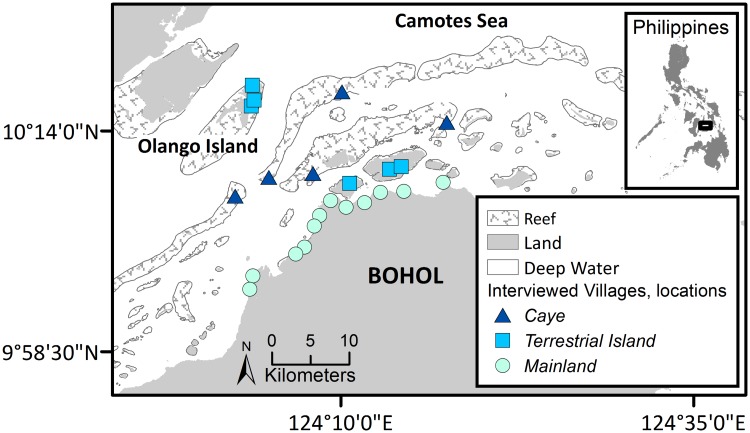
Map of the study area and 23 sampled villages in the Danajon Bank, Philippines, a biodiversity hotspot within the Coral Triangle. Symbols indicate the location of villages in the ecosystem.

We focused on small-scale fisheries in the Danajon Bank (Central Visayas, Philippines; 10°15’0’N, 124°8’0’E; Fig 1). This case study is representative of small-scale fisheries where there is a combination of low technology, high biodiversity, and a large number of fishers dependent on marine resources. Additionally, the spatial segregation of small-scale fisheries and industrial fisheries (see details below) provides a rare opportunity to explore the development and impacts of small-scale fisheries in isolation. The Danajon Bank coral reef ecosystem is characterized by large proportion of degraded habitats [38] and exceptionally low biomass of fish (e.g. demersal fish biomass 0.45 tons/sq km) [39]). The Danajon Bank sits off the northern and northwestern coasts of Bohol—a province that struggles with extreme poverty and minimal infrastructure [40], traits that are typical of places supporting small-scale fisheries. Fishing is a primary human activity influencing the Danajon Bank because of the high population density and lack of alternative livelihoods. The Danajon Bank lies very close to Cebu City, the second largest metropolitan area in the Philippines (with 2.8 million people). However, the Danajon Bank’s communities are rural and travel links to the city are weak.

Contemporary fisheries management in the Philippines emphasizes co-management, gear restrictions, spatial restrictions, and marine protected areas (MPAs) [34,41]. In the Philippines, small-scale fishers are legally known as ‘municipal’ fishers. To participate in these fisheries, boats must weigh 3 gross tons or less. Most fishers use outrigger canoe style boats, but engine-use has increased from approximately 20% of boats in 1960 to 50% of boats in 2010 [42]. Small-scale fisheries in the Danajon Bank are multi-gear, multi-species, and effectively open-access [42,43]. Filipino laws have given small-scale fishers exclusive rights to fish in inshore waters < 15 km from coasts since 1991. The entire Danajon Bank falls within inshore waters. Despite ongoing management efforts, heavy fishing pressure, destructive fishing, and high population densities continue to apply huge pressures on marine ecosystems [35,43]. Our study focused on an 800 km^2^ section of the central Danajon Bank. This area spanned five municipalities, two provinces, and a gradient from inshore turbid waters to offshore clear waters.

### Eras of fisheries governance

Six decades were examined in our study and partitioned into governance eras based on extensive review of government fisheries documents, legislation and development projects, and other published and online sources. We focused on the three aspects: (i) level of organization and power, (ii) aims and values of fisheries legislation, and (iii) aims and values of development funding. Each was evaluated according to its management tools, institutional formation, as well as underlying principles and values, thereby providing information on different aspects of governance [20]. Recent participation in co-management was characterized by: (i) presence of community-established marine protected areas (MPAs) and (ii) participation in fisher organizations (details in Fisher Interviews).

### Fisher interviews

To document temporal changes in fishing activities, we drew on LEK by conducting semi-structured interviews between July 2010 and April 2011 (n = 391 fishers) in 23 fishing communities (approximately 50% of the communities in the study area; S1 Table). LEK integrates the customary knowledge, practices, and beliefs of communities regarding the local environment and their relationship with it [44,45]. We stratified fishing communities by their location (Coastal, Terrestrial Islands, Cayes; Fig 1) [46] to ensure we captured representative samples of fishing gears adapted to local environmental differences (e.g. mangroves vs. reefs). We then proportionally sampled randomly-selected communities from within each group using a randomly-generated numbered list. We obtained written consent from municipal mayors, in accordance with Philippine laws, and oral consent from elected village officials, as well as every individual respondent. When oral consent was given by respondents, it was recorded on consent forms. Oral consent was most appropriate culturally and preferable due to the low levels of education and literacy among some participants. Research methods, including consent procedures, were approved by the University of British Columbia’s Human Behavioural Research Ethics Board (H07-00577).

Within each participating community, we identified fishers using village-level census data. Throughout the Philippines, census records are collected regularly by community health workers. Where occupational data was incomplete, outdated, unclear, or had questionable accuracy (e.g. 25 year olds listed as high school students), health workers provided missing information. For example, health workers identified those fishers who had moved or passed away. We used village census records rather than municipal records of fishers because we found that municipal data severely underestimated the number of all people who fished. Census occupational data also provide a conservative estimate of fishers because these records do not include children or women who fish [47]. We did not attempt to correct for this bias.

Within each fishing community, we randomly sampled 7% of full-time and part-time fishers. To obtain longer time series of information, we stratified fishers by age, focusing on fishers born before 1981 whom we estimated had been fishing for at least 15 years. This method may underrepresent recent trends influenced by young fishers, but prioritizes a long time-series of fishing. Interviews were conducted in the local language (Cebuano) by two Cebuano-fluent local research assistants.

During interviews, we systematically reconstructed the history of the respondent’s fishing practices, as far back as the respondent could remember, using three steps [48]. First, we constructed personal timelines for each respondent which helped fishers link reported changes in fishing practices with important dates (e.g. age started fishing; date of children’s births) [49]. Second, we determined which years each respondent fished in the Danajon Bank (either full- or part-time), any periods where he stopped fishing, as well as any monthly or migratory patterns in his fishing. Also, the days per year that a respondent fished in our study area was used to quantify individual fishing effort (number of days fished per year by individual fishers). As some respondents migrated to other provinces for several months a year, we excluded any fishing activities outside of the Danajon Bank from this study. Third, we made timelines for each fishing gear that each respondent used in our study area. For each gear, we recorded the years the respondent fished with the gear and any details he shared about the fishing gear (e.g. catch). During interviews we validated the consistency of fisher responses using internal triangulation. We asked about key information during multiple questions, which allowed us to cross-check the consistency of fishers’ responses (i.e. triangulation). This enabled us to correct inconsistent or illogical answers during interviews [50]. We focused on fishing gears and effort rather than catches as these aspects of fishing tend to be relatively consistent over time. This consistency makes gears and effort easier to recall than variable aspects of fishing, such as catches [51,52]

### Indicators of co-management participation

Fishers’ organizations are one official component of Philippine local co-management structure, as outlined by the 1998 Fisheries Code (RA 8550). We sought to evaluate the presence of these organizations, and the level of fisher engagement. We asked a subset of respondents (n = 256 fishers) whether fishers’ organizations existed in their communities, whether they participated in the fishers organizations, and the year that they had joined the organizations. We also used expert interviews with local community organizers to confirm information about when fisher organizations were established. Since MPAs are a second component of local fisheries co-management (RA 8550), we identified which communities had established MPAs through expert interviews, websites of municipal governments, and published literature [53].

### Fishing gear classifications

For gear classifications, we developed a database based on information derived from surveys as well as from key informants, our own field knowledge, and published literature on fishing gears [12,17,32,54–61]. We assigned each specific gear to one of eight general gear classes that we set to fit the small-scale fisheries context (Table 1) [48]: hook & line, nets, diving, traps, blast fishing, poison, fish corrals, and gleaning. For example, hook-and-line methods include gears with various numbers of hooks, lines, and weights.

**Table 1:** The eight general classes of fishing gears and some examples of specific gears and their classification in intensive gear categories.

General gear class	Specific gear	Intensive Categories			
		Non-selective	Active	Destructive^a^	Illegal^b^
hook & line	hand line, 1 hook				
	benthic longline				
nets	bottom set gillnet	X			
	encircling gillnet	X	X		1998 (if automated when deployment from boats)
diving	spear	X	X		
	crowbar (Cebuano: kay-kay)	X	X	breaks corals	1998
traps	large fish traps				
	crab traps				
blast fishing	fertilizer bomb	X	X	shatters corals	1932
poison	squirt bag	X	X	kills corals	1932
	in traps	X		kills corals	1932
fish corral	v-shaped weir				
gleaning	hand	X	X		
	machete	X	X	breaks corals	1998

Intensive categories indicate if the gears are: active, destructive, non-selective, and/or illegal.

^a^Brief descriptions of habitat impacts are included for destructive gears.

^b^For illegal gears we provide the year that the gear became illegal and any conditions about the legality, if relevant.

In coral reefs, fishing gears span a range of intensities, from hand-reeled lines that catch one fish at a time (handlines), to homemade explosives that kill entire schools of fish and shatter adjacent corals (blast fishing). We define intensive fishing gears as those with a high catch efficiency, a great magnitude of force, and/or a relatively low selectivity. In this way, intensive gears have higher catchability and are more efficient than non-intensive gears. However, intensive gears may cause more extensive changes to the ecosystem (e.g. via damaging habitat, catching juveniles). Although illegal fishing gears are not inherently intensive, the majority of illegal gears in the Philippines are also intensive. Thus we group illegal gears with intensive gears, while recognizing that this relationship may not be relevant in other contexts. Fishing intensification differs from agricultural intensification [62], because there are no inputs that influence production (e.g. fertilizer) and no opportunity for selecting high yield varieties because fishers target available species. Thus there is an increase in extractive effort, without a corresponding effort to support the systems’ productivity. Since the intensity and impacts from small-scale fishing gears have rarely been quantified, assessing changes in gears provides a process for evaluating the potential impacts of fishing (but see, for example, [17,18,61] for small-scale fisheries).

We assigned the 93 specific fishing gears into four pairs of intensive/non-intensive categories: destructive/non-destructive; active/passive; non-selective/selective; and illegal/legal [48]. Destructive gears damage habitats (e.g. blast fishing). Actively moving gears heavily exploit marine life (e.g. trawling) and raise concerns in fisheries conservation (for example, [63]). Non-selective gears catch juveniles and non-target species (e.g. small-mesh nets) [18]. Illegal gears may be intensive or may be prohibited for other reasons (e.g. dangerous for fishers), and are defined by regulations rather than by impact. Some gears were illegal throughout the study period (e.g. blast fishing), while other gears became illegal during the study period (e.g. fishing with a crowbar which damages habitat and thus became illegal under the 1998 Fisheries Code). We placed each specific gear in only one category within each pair (i.e. destructive *or* non-destructive), but a fishing gear could belong to more than one intensive category (e.g. beach seines are destructive, active, and non-selective).

### Data analyses

#### Trends in individual and aggregate fishing gears

First, we calculated the mean number of specific gears that individual fishers had used throughout their careers. Second, we evaluated how the mean number of specific gears that individual fishers used in a year changed over time. Third, we assessed how two metrics of gear diversity had changed over time for specific gears: gear richness and the Simpson's Index of Diversity [64]. Gear richness (G; hereafter diversity) was estimated as the total number of unique gears (*g*) used in a year (*t*)

$$G=\sum_{1}^{f}g_{t}$$ (1)

The Simpson's Index of Diversity (D) considers both gear richness and evenness by estimating the probability that two gears taken at random will represent the same gear. We estimated D where *N*_*t*_ is the total number of any type of gear used by all fishers in year (*t*) and *n*_*t*_ is the total number of gears of a particular type of gear used by all fishers in a year (*t*)

$$D=1-\frac{\sum_{1}^{G}n_{t}\left(n_{t}-1\right)}{N_{t}\left(N_{t}-1\right)}$$ (2)

For analyzing these gear changes over time, we divided the fishing timelines into the four governance eras and sampled gear-use during six randomly selected years from each era. The residuals from the Bartlett test comparing changes in in gear use and diversity were non-normally distributed, so we used Kruskal-Wallis Rank Sum tests to compare differences in gear use between governance eras [65]. We used a post-hoc Kruskal-Wallis Multiple Comparison test to determine which governance eras exhibited significant differences in gear use and diversity.

#### Trends in general, intensive and non-intensive fishing

To understand trends in fishing activities, we evaluated how three aspects of fishing developed over time and in relation to governance eras: (i) total fishing effort (cumulative number of days fished in one year by male fishers from the 23 participating villages); (ii) relative fishing effort (i.e. the proportion of total fishing effort allocated to each fishing gear in one year); and (iii) the proportion of fishers using various fishing gears in one year. We assessed the evolution of these three aspects of fishing for both the eight general fishing gear categories and the four (non-exclusive) pairs of intensive and non-intensive gears. Estimates for total fishing effort (days per year) assess the fishing effort for only the 23 participating communities.

For each category, we estimated effort and gear use using six steps. First, we used interview data to calculate the total annual fishing effort that all respondents fished as the sum of individual effort (days fished; *e*) by all respondents (*f*) in year (*t*)

$${\hat{E}}_{t}=\sum_{1}^{f}e_{ft}$$ (3)

Second, we evaluated the mean fishing effort in a year (${\overset{-}{E}}_{t})$ as the total days fished (${\hat{E}}_{t}$) in a year (*t*) divided by the total number of respondents (*f*) in year (t)

$${\overset{-}{E}}_{t}=\frac{{\hat{E}}_{t}}{f}$$ (4)

Third, for each year we divided individual respondents’ fishing effort among their actively used fishing gears. Fourth, we estimated gear-specific effort as the sum of effort (*e*) by individual respondents (*f*) using each gear (*g*) in each year (*t*)

$${\hat{E}}_{gt}=\sum_{1}^{f}e_{fgt}$$ (5)

Fifth, we estimated the relative (percent) fishing effort allocated to each gear (*g*) during each year (*t*) as

$$RE_{gt}=\frac{{\hat{E}}_{gt}}{{\hat{E}}_{t}}\;100$$ (6)

Sixth, we estimated the total fishing effort allocated to each gear by multiplying (i) proportion of effort allocated to that fishing gear by all fishers (*RE*_*gt*_), (ii) mean fishing effort (${\overset{-}{E}}_{t})$, (iii) the population of participating villages (*V*_*t*_), and (iv) the proportion of the population (*P*_*t*_) who fished during a year (*t*) (adapted from [8] to include effort and time)

$$E_{gt}=RE_{gt}\;{\overset{-}{E}}_{t}\;V_{t}P_{t}$$ (7)

Lacking other data, we assumed that the proportion of the population that fished (*P*_*t*_) was static through time[48]. Using proportions and estimates rather than raw sums of effort allowed us to compare across the study period, despite the temporally-varying sample sizes of fishers[48].

After obtaining estimates of total fishing effort, relative fishing effort, and the proportion of fishers using various fishing gears, we analyzed changes over time using generalized least square models[65]. The explanatory variables were governance era and year. We assumed governance era accounted for changes in fishing regulations and governance structures. Year may indicate changes in pressures on the ecosystem, underlying changes in the abundance of species, or supporting ecological processes. Models incorporated temporal auto-correlation using a corARMA auto-correlation structure and used governance era as a variance covariate to allow for the heterogeneity of variances within governance eras [65]. Due to high variance in the first decade, we restricted analyses of fishing activity to the period 1960–2010. We conducted all analyses in R 3.3.1 (R Core Team 2016) using packages dplyr, pgirmess, MuMin, and nlme.

## Results

### Eras of fisheries governance

In the past 60 years, fisheries governance in the Philippines has changed substantially. We split those changes into four eras, to which we have assigned periods and names: *Traditional Governance with Limited State Involvement* (1950–1971); Productivity (1972–1985); Decentralized Governance (1986–1997); and Co-management (1998–2010). These eras were distinguished by differences in governance priorities (e.g. production, sustainability) and relative influence of local vs. state institutions in the implementation of fishing policies (Table 1).

#### Traditional governance with Limited State Involvement Era (1950–1971)

The Traditional Governance Era was characterized by local tenure and minimal intervention from the national government. At the local level, traditional governance included the rights of some households to fish corrals, fish shelters, and mangrove trees [57]. These tenured households also held the right to designate access to others [57]. More extensive local governance may have existed during this time, but is not well documented. At the national level, fisheries policies were set by the central government, and little attention was paid to small-scale fisheries [34,66]. Resources officially fell under state ownership and were open access, although local municipalities were able to grant licenses to commercial fishers in the first 5.5 km of coastal waters [67]. Beginning in 1932, three destructive gears were banned nationally (blast, poison, electro—fishing), demonstrating some attention to conservation. The ban on these three types of destructive fishing remained in place throughout the period under study.

#### Productivity Era (1972–1985)

In the Productivity Era, fisheries governance remained focused at the national level, with a series of legislation and development programs emphasizing increasing catches and maximizing resource extraction [34,66]. For example, in 1975 Presidential Decree 704 emphasized development, productivity, and building fisheries exports (Table 2). The decree paid little attention to conservation, but maintained existing gear bans and added restrictions on fine mesh nets. During this period, fishing subsidies allowed fishing effort and catches to increase (e.g. World Bank credits for motorized engines) [68,69]. This focus on maximizing resource extraction was consistent with global practices at that time [22]. The first signs of overfishing and habitat declines appeared during the Productivity Era [69]. Most records of emerging troubles, however, are from the Manila region and there is less documentation of trends in the central Philippines.

**Table 2:** A brief overview of four eras of Philippines fisheries governance.

Era	Years	Legislation	Major Components
Traditional	1950^a^ –1971	Admin. Code, 1917	National small-scale fishing regulations
			Allows municipal council to grant fishing rights
		Fisheries Act, 1932	Closed seasons, some access regulations
		Republic Act, 428	Limited commercial fishing inshore
			Three destructive methods banned (blast fishing, poison fishing, electro-fishing)
			Marine protected areas established at the municipal level
Productivity	1972–1986	Republic Act, 6451	National small-scale fishing regulation
		Fisheries Decree, 1975	Emphasis on development and productivity, with little attention to conservation
		Presidential Decree, 704	Ban on fine mesh nets, poison, blast, electro fishing
			Limit municipal boats < 3 gross tons
			Municipalities issue fishing licenses, but require national approval
			Municipal waters set at 3 nautical miles from shore
			MPAs set by national government
		Presidential Decree, 1219	Gathering coral banned
Decentralized	1986–1997	Local Gov. Code, 1991	Municipal fishing regulation of small-scale fisheries
			Prohibited commercial fishing within 15 km of shore (municipal waters)
Co-management	1998–2010^b^	Fisheries Code, 1998 (Republic Act, 8550)	Co-management of small-scale fisheries formalized
			Responsibilities shared by local institutions, stakeholders, NGOs, and national governments
			Destructive gears banned
			Some active gears restricted
			Marine protected areas established at the municipal level
			Target of protecting 15% of coastal waters
		Philippine Marine Sanctuary Strategy (2004)	Target of protecting 20% of coral reefs by 2020.

^a^The period under study began in 1950, but the fisheries laws in place at that time were from earlier legislation

^b^Following this study, the 1998 Fisheries Code was updated (RA 10654, 2015)

#### Decentralized Era (1986–1997)

The Decentralized Era, which began after the Marcos dictatorship lost power in 1986, saw the national government cut funding and devolve responsibility for management of natural resources—and other sectors (e.g. healthcare)–to local governments [70]. This structural transformation followed global trends in the 1980s and early 1990s, a time when decentralization was considered the best approach for economic development [71]. The 1991 Local Government Code formalized decentralization by shifting management of most natural resources from the national to the municipal level. As a result of this devolution of governance, municipal governments were given the responsibility of managing small-scale municipal fisheries [34]. Furthermore, commercial fishing was prohibited within 15 km of coasts, though there was ongoing debate about how these boundaries were defined. There were no significant changes to fishing gear regulations during this Era.

#### Co-management Era (1998–present)

The Co-management Era began when sweeping fisheries legislation (Republic Act 8550:1998 Fisheries Code) strengthened the rights of small—scale fishers to exclusively use inshore waters up to 15 km from shore and established new institutional arrangements within the previously decentralized government. Small-scale fishers’ rights to inshore waters were set both through moderately effective national regulations banning all commercial fisheries and through largely unenforced regulations restricting fishing to an individual’s home municipality [42,72]. New institutional arrangements outlined in the fisheries code were designed to facilitate co—management. For example, the Fisheries Code explicitly required local participation by mandating that fisheries governance be shared among local governments, fishers’ organizations, and NGOs. During the Co-management Era, some fisheries regulations were still set at the national level (e.g. some gear regulations) [66,73]. However, many aspects of small-scale fisheries governance, including the implementation of national and local policies, were conducted at the municipal level [34,66,67,74]. Existing research has found that, in some regions, the Co-management Era had stronger local capacity and participation than the Decentralization Era because local capacity and fisher involvement in management was gradually strengthened through the support of development programs and NGOs [75,76]. However, other in areas fisheries management remained a low government priority [71]. After the field work for this study was completed, the Philippines revised its Fisheries Code. The revisions (Republic Act 10654, 2015) contain a more explicit emphasis on ending illegal, unreported, and unregulated (IUU) fisheries and promoting conservation.

Under the 1998 Fisheries Code, municipalities are responsible for meeting nationally set targets for establishing MPAs to protect 15% of coastal waters (1998 Fisheries Code). Additionally, the country has committed to protecting 10% of reef areas by 2020 (Philippine Marine Sanctuary Strategy, 2004). In the Danajon Bank, the process of establishing MPAs has largely been driven by fishing communities rather than municipal governments [46]. To date, locally-enforced no-take MPAs have been established in over 40 locations [53].

The Co-management Era, beginning in 1998, had several implications for fishing gears. First, all gear that damaged coral reefs, seagrass, mangroves or other marine life habitat became illegal. Second, active gears became illegal in municipal waters, although this restriction focused on gears deployed by boats (e.g. trawl, purse seines, drift gill net, tuna longline) that were deployed by boats. Other active gears (e.g. diving), however, remained permitted. Third, small-mesh nets (< 3 cm mesh) were prohibited in most cases, but remained legal for some species.

### Indicators of co-management participation

When we examined proxies for governance participation during the Co-management Era, we found that 74% of villages had established fishers’ organizations and 19% of fishers were members of fishers’ organizations, with much variation in participation (0–80%) among villages. In all, 70% of study villages had locally implemented MPAs. Villages established MPAs between 1996 and 2007. All but one MPA were established after 1998, the first year that local governments had the autonomy to establish MPAs.

### Trends in individual and aggregate fishing gears

During the four governance eras, fishers reported using 93 fishing gears, which we categorized into eight general classes: diving, nets, traps, hook-and-line, gleaning, corrals, poison, and blast fishing (Table 2) [48]. Some gears were designed to target specific species (e.g. jigs for octopuses) while other fishing methods aimed to catch anything of value (e.g. skin diving).

**Fig 2:**
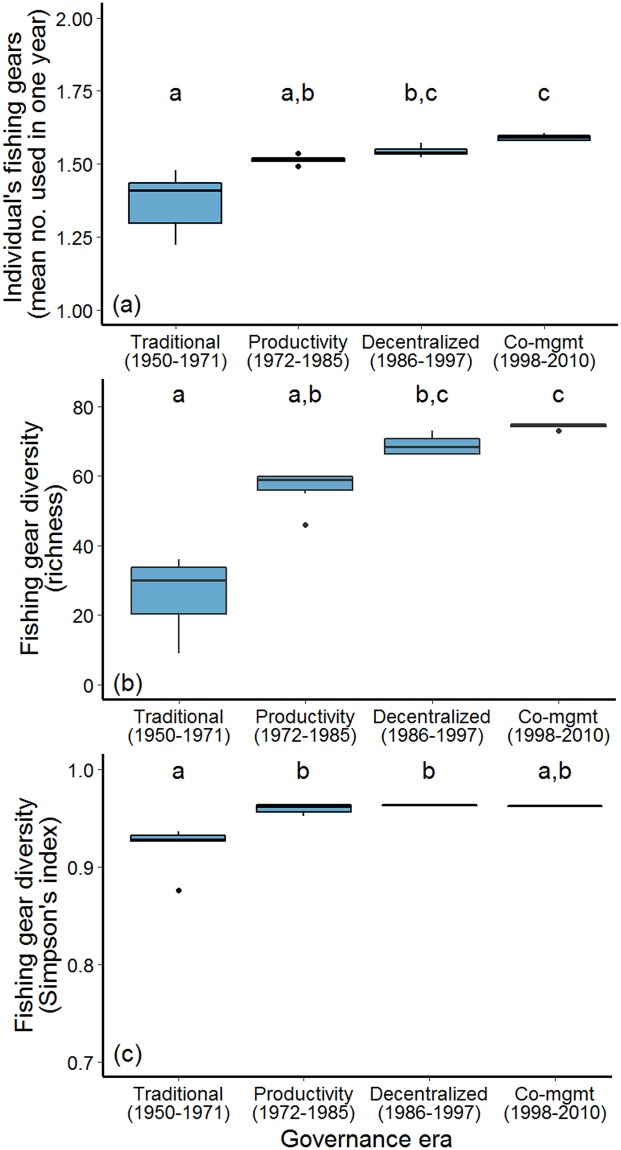
Changes in fishing gears during four eras of fisheries governance (1950–2010) (n = 391 respondents). (a) Mean number of small-scale fishing gears used by individual fishers. (b) Richness of small-scale fishing gears (i.e. total number of gears used by all fishers). (c) Simpson’s Index of Diversity of small-scale fishing gears used by all fishers. Fishing gears were classified as 93 specific gears and six randomly selected years were sampled during each Governance Era. Letters denote significant differences in gear use between Governance Eras at p < 0.05 as indicated by a Kruskal-Wallis Multiple Comparison post-hoc test. This change was largely due to the growing number of fishers.

Over their fishing careers, individuals used a mean of 2.47 (± 0.07 SEM) gears (range: 1–7 gears over their careers). Individual fishers used anywhere from 1–6 fishing gears in a single year. The mean number of gears used by individual fishers in a given year increased over time (Kruskal-Wallis X^2^ = 20.94 df = 3, p < 0.001), but remained below two in all eras (Fig 2A). Gear diversity (richness), the number of gears cumulatively used by all respondents, ranged from 9–75 gears per year. Rapid growth in diversity was evident from the 1950s onwards, peaking during the Productivity Era (Fig 2B; Kruskal-Wallis X^2^ = 21.62, df = 3, p < 0.001). High Simpson’s Index of Diversity was found across all eras, suggesting that most gears were used by a relatively small proportion of fishers (Fig 2C; range = 0.87–0.96, Kruskal-Wallis X^2^ = 14.27, df = 3, p = 0.003).

### Trends in individual and aggregate fishing

**Fig 3:**
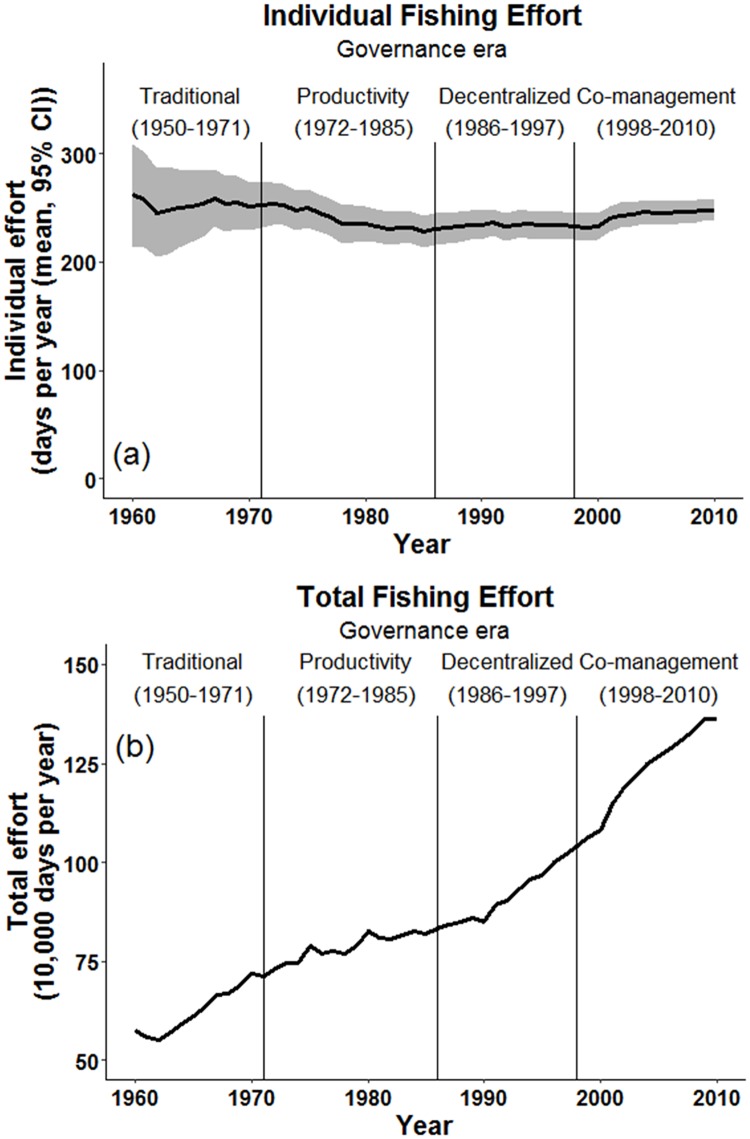
Long-term changes in fishing effort in the Danajon Bank, Philippines. (a) Mean individual fishing effort (95% CI). (b) Estimated total fishing effort (total number of fishing days by all fishers in 23 participating villages).

From 1960–2010 mean individual fishing effort remained fairly steady (range: 228–262 days per year; Fig 3A). In contrast, total fishing effort increased 2.4 fold, from approximately 576,000 fishing days per year in 1960 to approximately 1,363,000 days per year in 2010 (Fig 3B) [48].

### General fishing gears

**Fig 4:**
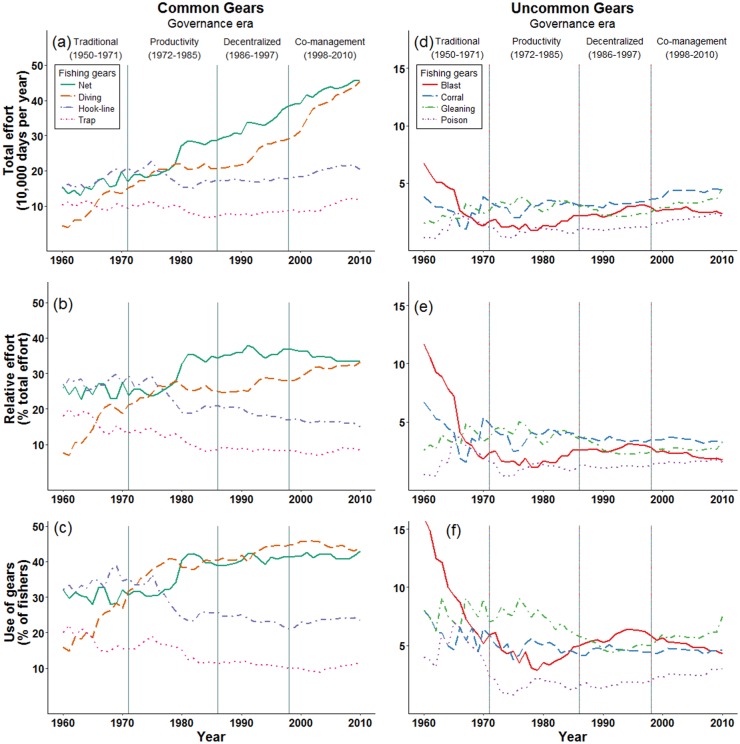
Long-term changes in fishing activities by multi-gear small-scale fisheries in the Danajon Bank, Philippines. (a-c) Changing use of the four most common fishing gear categories. (d-f) Changing use of four relatively uncommon fishing categories. (a,d) Estimates of total fishing effort by fishers from the 23 study villages. (b,e) Relative fishing effort. (c,f) Percent of fishers using these categories of fishing gears during any time in a year.

We found oscillations in the dominant fishing gears for both common and uncommon gears (Fig 4).

Initially, hook & line gears and nets had the highest total fishing effort, and this gradually transitioned to dominance by nets and diving.

- The total effort of nets increased 2.9 fold over time (Fig 4; S2 Table), while the proportion of fishing effort and the proportion of fishers using nets exhibited 1.2 and 1.3 fold increases, respectively (Fig 4; S3 and S4 Tables). The greatest growth in the total effort and relative effort of nets occurred during the Productivity Era when nets exhibited a 1.5 fold increase in both metrics.
- Total and relative effort spent diving increased significantly over time, although increases varied among governance periods (Fig 4; S2 and S3 Tables). Diving methods exhibited fairly steady total effort throughout the Limited and Productivity Eras, with fishing effort levels sitting at approximately 200,000 days per year (S2 Table). From the Decentralization Era through the Co-management Era (1986–2010) there was a 2.2 fold increase in total diving effort. The proportion of fishers using diving also increased over time, but at different rates during different governance eras. There was a strong increase in the proportion of fishers using diving throughout the Limited and Productivity Eras (2.5 fold increase). Since the mid-1980s, however, the proportion of fishers using diving has only increased slightly (1.1 fold increase).
- During the study period, both hook & line and trap gears exhibited moderately steady levels of total effort (1.4 and 1.1 fold increases, respectively), but their relative effort declined to 57% and 47% of initial levels (Fig 4; S2 and S3 Tables). In particular, the relative effort and proportion of fishers using hook & line gears declined during the Productivity Era.

The four uncommon fishing gear categories showed highly variable patterns. In most years, these four gears each contributed to less than 6% of the total fishing effort.

- Blast fishing initially comprised approximately 12% of total fishing effort. During the Limited Governance Era (1960–1971) blast fishing declined to a quarter of its initial levels, comprising less than 2% of total fishing effort by the early 1970s (Fig 4; S2 Table). During the middle of the Productivity Era, the use of Blast fishing began slowly rising again—a pattern which continued until the middle of the Co-management Era. In 2010 this highly damaging gear was used by a low share of all fishing effort (1.7%). However, this still amounted to more than 23,000 fishing days per year in 2010.
- Total effort by poison fishing was highly variable during early periods, and then exhibited a 1.8 fold rise in total effort under Co-management (Fig 4). In 2010 the total effort of poison fishing was similar to total effort of blast fishing.
- Total gleaning effort was initially variable and then increased 2.15 fold from the mid-1990s to 2010 (Fig 4). Currently, we estimate that total gleaning effort is over 40,000 days per year.
- Fish corrals were used with a relatively steady amount of total fishing effort (Fig 4; S2 Table). They increased 1.1 fold in total effort over time, and in 2010 were used approximately 44,000 days per year. In contrast, the relative effort of fish corrals decreased to 48% of their initial levels and the proportion of fishers using fish corrals decreased to 57% of initial levels (S3 and S4 Tables).

### Intensive, non-intensive, and illegal fishing gears

The relative distribution of intensive and non-intensive categories was consistent over time (Fig 4). We report a steady rise in total effort by all intensive fishing gears over time. From 1960–2010 total fishing effort increased for destructive gears (3.4 fold increase), active gears (3.0 fold increase), and non-selective gears (1.5 fold increase). After the Decentralization Era began (1986 onward), the percent of fishing effort allocated to these three intensive gears and the proportion of fishers using these gears remained relatively steady. However, this steady pattern of relative fishing effort did not translate to total fishing effort. During the Decentralization and Co-management Eras, total fishing effort using these three intensive gear categories continued to increase.

**Fig 5:**
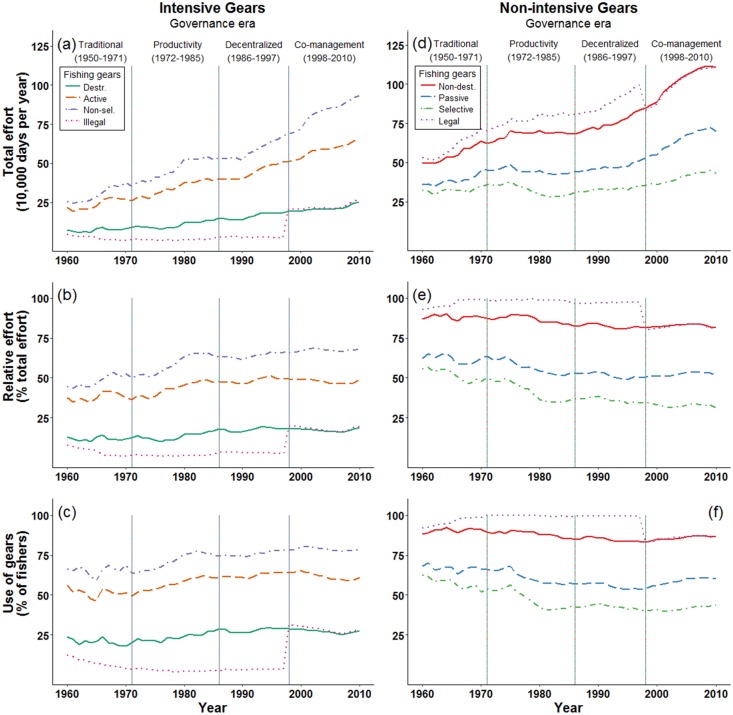
Long-term changes in fishing activities by multi-gear small-scale fisheries in the Danajon Bank, Philippines. (a-c) Changing use of four (non-exclusive) categories of intensive fishing gear. (d-f) Changing use of four (non-exclusive) categories of non-intensive fishing gears. (a,d) Estimates of total fishing effort by fishers from the 23 study villages. (b,e) Relative fishing effort. (c,f) Percent of fishers using these categories of fishing gears during any time in a year.

During our study period, total effort by illegal gears declined by a factor of 1.7 until 1998 (Fig 5; S2 Table). After the 1998 Fisheries Act, many existing gears became illegal. Thus the use of illegal fishing gears (total effort, relative effort, and the proportion of fishers) using illegal gears exhibited an 8.4 increase in 1998. Following this change, total effort by illegal gears remained relatively steady. However, in 2009 and 2010 total effort by illegal gears increased 1.2 fold from approximately 231,000 to 265,000 days per year.

Non-intensive gears also increased during the period under study, but these increases were more gradual than for their intensive counterparts. From 1960–2010 total fishing effort increased for non-destructive gears (2.2 fold increase), passive gears (2.0 fold increase), and selective gears (1.3 fold increase) (Fig 5; S2 Table). The proportion of fishers using non-intensive gears declined slightly over time, although these changes were relatively small (non-destructive and passive = 1.1 fold decrease; non-selective = 1.4 fold decrease) (S4 Table).

## Discussion

Our rare and integrated analysis of small-scale fishing over six decades found that fishing gears have diversified, intensified, and that total fishing effort and use of gears with high ecological impacts has greatly increased. As the number of fishers grew, there was a corresponding increase in the diversity of fishing gears. Despite the systemic increase in the total number of gears used, most individual fishers used fewer than three gears over their entire careers and only one or two gears each year. We found individuals fished a relatively consistent number of days per year throughout the period under study, but that total fishing effort across the study area increased 240%. We note that fishing effort is not distributed evenly in space, and there is evidence that some locations experience substantially greater increases in effort than those documented here [42]. Fishers increased their total effort using nets and diving, with other fishing gears showing less pronounced changes in total effort over time. When evaluating gears according to intensity, we documented increasing use of non-selective, active, and destructive gears. Mounting use of these intensive gears outpaced the growth of their non-intensive counterparts (i.e. selective, passive, and non-destructive gears). This burgeoning of gear diversity, effort, and intensity, occurred before the mid-1980s, underscoring the value of a long-term perspective for understanding the development of small-scale fisheries.

### Benefits of gear diversification and persistence

This diversification of fishing gears may benefit fishers in four ways. First, targeting many species through gear diversification can improve the economic value of catches. For example, catching a large number of species benefited Kenyan fishers by increasing catch-per-unit-effort (CPUE) and mean trophic levels of catches [77]. Second, new gears potentially enable catch of new species, a necessary substitution when original targets decline. In the central Philippines, for example, substitution allowed continuation of fishing after apparent extirpation of formerly targeted species [78]. Additionally, catching new species can allow fishers to take advantage of emerging opportunities, such as access to global markets for species such as seahorses, sea cucumbers, and abalone [15,79]. We infer that the shift we documented from dominant hook-and-line fishing effort to dive fishing effort indicates a corresponding shift in targeted species from primarily fishes (caught by hook-and-line [47]) to a mix of fish and invertebrates (caught by divers [47]). In the Danajon Bank, shifts in targeted species has been observed by previous research [72], but not linked to corresponding changes in fishing gears. Third, new gears can provide a competitive edge for catching previously-targeted species in novel ways (e.g. new habitats; new life history stages). Fourth, modifications can improve gear efficiency and gear longevity. In European fleets, for example, small, step-wise modifications in gears during the last century and large changes in gears over the past two hundred years led to significant increases in catchability [13,80]. Here, respondents described how modifications of gears can improve gear longevity by incorporating durable materials (e.g. shifts from bamboo to plastic materials for traps). We infer that the increasing numbers of gears, combined with increasing distance travelled [42], helped offset growing total fishing effort. In contrast, high fishing effort resulted in decreasing gear diversity in a Kenyan small-scale fishery where accelerated use of destructively efficient nets diminished the use of other gears [77].

We documented strong inertia of familiar fishing gears, highlighting the long-term implications of governance priorities and policies. We found the greatest relative increase in active and non-selective gears during a period when Filipino governance emphasized resource exploitation to meet economic goals including, putatively, food security (i.e. Productivity Era) [22,71]. During this period, development programs in the Philippines provided fishers with more efficient gears [69], which then lingered for the long-term. In this case, the use of active and non-selective fishing gears persisted for decades, even after Filipino governance priorities shifted towards sustainable fishing [32,81]. The 'stickiness' of these changes is evidence that policies created under the ethos of one era can have a lasting, and possibly unintended influence on future practices [82]. Use of damaging gears may persist for many reasons including familiarity, poverty, social norms, lack of knowledge about the link between destructive gears and diminishing catches, and prohibitive startup costs or time for learning new methods [83,84].

Where poverty is common, households frequently diversify their sources of revenue [85] often involving different livelihoods (e.g., gold panning, agriculture, forestry and fishing). It’s conceivable that diversifying the methods used to maintain a single livelihood activity, such as fishing, could achieve outcomes similar to diverse livelihood portfolios. We found, individually, each fisher used relatively few gears, a choice with implications for their livelihood portfolios. Where livelihood opportunities are sparse, use of several gear types might arguably reduce a fisher’s dependency on one group of species, as well as protect fishers from ecological [12,86] and market variability [57,85]. Yet fishers on Danajon Bank did not adopt this strategy, instead relying on few gears (1–2) at any given time. This result supports previous findings that fishers may not perceive benefits from diversifying their gears [87], or that fishers can be limited by the skills or capital investment required for new gears [57]. The corollary is that fishers may perceive or acquire greater benefits by using existing gears [88] or by diversifying into other livelihoods beyond fishing [89].

### Quantifying historical fishing activities with LEK

A lasting ability to manage resources requires historical context for current conditions, relying on estimate of long-term trends. In data-poor situations, such estimates must rely on diverse data sources [9]. Our research confirms the role of local knowledge in developing a quantitative understanding of changes in resource extraction. Our work collected local knowledge in a technically rigorous fashion (e.g. through randomization of villages and respondents), providing information otherwise unavailable on six decades of fishing practices and their impact. We acknowledge that local knowledge has limitations, such as the loss of details over time. Thus, we assume that fishing estimates from the earlier years under study (e.g. Traditional Governance Era) are less precise than later eras. This variance was reflected in the error measurements from that period. To some extent these limitations can be mitigated with appropriate sampling and survey designs [49], such as those we used. We recognize that young fishers (born after 1981), for whom we have a relatively smaller sample, could have made very different choices regarding type of fishing gears and frequency of use. Furthermore, fishers who stopped fishing by the time of the survey may have been under-represented. Nonetheless, our novel approach still provides useful information and is flexible enough to be applied to other resource management contexts.

### Implications for fisheries management

Reducing fishing effort in small-scale fisheries requires striking a careful balance between ending overexploitation of ecosystems and building adaptive capacity within fishing communities. Our investigation of historical patterns is relevant to the challenges facing long-term fisheries management for a variety of reasons. Our results revealed large increases in total fishing effort by non-selective, active, and destructive fishing gears. As fishing effort is highly concentrated in space, there is evidence that there was a significantly higher growth of fishing effort in popular fishing locations [42]. This knowledge of past change supports establishment of appropriate fishing targets and planning for future changes a system can accommodate [90]. For example, scaling back active and non-selective fishing methods to 1980 levels of effort would require a 56% reduction in fishing effort. Such reduction is similar to that recommended by other evaluations of small-scale fishing in the Philippines [81].

Second, it will be both important and rather complicated to shift fishers’ away from intensive fishing practices that may be well-entrenched. The “stickiness” of familiar fishing gears we observed also indicates that use of non-intensive gears would persist once established. The increasing prevalence of intensive fishing gears, however, combined with the large increases in total effort, potentially results in more ecological impacts than effort increases solely using non-intensive gears [91]. Remarkably, there are few studies that focus on the effects of changing small-scale fishing gears in coral reefs, but existing evidence does point to benefits from eliminating intensive gear. For example, Kenyan fishing grounds that excluded beach seines—a destructive, active, and non-selective gear—had higher catches than sites where such intensive gears were used [91]. Benefits were short-lived, however, later reduced by long-term increases in overall fishing effort. Successful gear management could emphasize reductions in effort and intensive gears as synergistic goals. Any shift towards non-intensive gears would involve dual approaches encouraging existing fishers to switch gears and/or ensuring new fishers adopt less damaging gears. As the Philippines implements recent revisions to the 1998 Fisheries Code, prioritizing ‘conservation, protection and sustained management of the country's fishery and aquatic resources’ [23], managers should consider how to incentivize fishers to discard intensive gear and dissuade new fishers from their adoption.

Third (and a corollary of the second), managers should be vigilant regarding new emerging fishing gears. The progressive diversification of gears we observed occurred through both technology creep (modification of existing gears) [13], as well as development of entirely new gears [80]. Adaptions in net materials and mesh size substantially effect catches [18], but this facet of fishing gears was not consistently available from our interview data. A forward-looking advantage of the Philippines’ broad fishing laws (e.g. prohibition of all habitat damaging gears) is that it encompasses new gears with undesirable externalities. Pro-active restrictions on development of new, intensive gears through regulations based on collateral impacts (e.g. habitat damage, low selectivity), rather than regulations focusing on specific gear types, could greatly aid fisheries management. Large marine protected areas might also provide refuges to offset the long-term environmental costs of damaging and non-selective gears [80].

### Conclusions

Despite growing commitments to fostering sustainability in small-scale fisheries [10], remarkably, little research has evaluated long-term changes in small-scale fisheries [11]. Our quantitative assessment of historical fishing practices targets this critical information gap and provides valuable advice for establishing sound methodologies to meet this goal. We determined that from 1950–2010, fisheries diversified, intensified, and increased in total fishing effort especially for intensive fishing gears. The priorities of fisheries governance were evident in the strong uptake of active and non-selective fishing gears during the Productivity Era. In many other cases, however, the fingerprint of governance was largely absent. Through this research, we demonstrate the fundamental role of fisheries policies and growing human populations in influencing changes in fishing effort and gears. This grounded historical understanding can provide direction and targets for ongoing efforts to reduce the effects of overfishing [5].

## Supporting information

S1 Table Summary information about 23 fishing communities in the central Danajon Bank, Philippines including population sizes, sample sizes, and the presence of fishers’ organizations and MPAs. (PDF)

S2 Table Summary of GLS model statistics for changes in the total fishing effort (fishing days per year by fishers in participating villages) allocated to gears in the central Danajon Bank, Philippines (1960–2010). Full models tested the effects of year and governance period, and their interaction on total fishing effort by each category of fishing gear. Models considered changes in the use of eight general classes of fishing gears and four pairs of intensive/non-intensive gear categories.
(PDF)

S3 Table Summary of GLS model statistics for changes in the relative fishing effort (percentage of fishing days per year) allocated to gears in the central Danajon Bank, Philippines (1960–2010). Full models tested the effects of year and governance period, and their interaction on relative fishing effort by each category of fishing gear. Models considered changes in the use of eight general classes of fishing gears and four pairs of intensive/non-intensive gear categories.
(PDF)

S4 Table Summary of GLS model statistics for changes in the proportion of fishers using various fishing gears in a year in the central Danajon Bank, Philippines (1960–2010). Full models tested the effects of year and governance period, and their interaction on the percentage of fishers by each category of fishing gear. Models considered changes in the use of eight general classes of fishing gears and four pairs of intensive/non-intensive gear categories.
(PDF)
