# Supplementary material for: Shifting gears: Diversification, intensification, and effort increases in small-scale fisheries (1950-2010)
Source: PLoS One. 2018 Mar 14;13(3):e0190232. doi: 10.1371/journal.pone.0190232 (PMC5851533; doi:10.1371/journal.pone.0190232)
Supplement: S1 Table — (PDF) [file pone.0190232.s001.pdf]

Jennifer C. Selgrath, Sarah E. Gergel, and Amanda C. J. Vincent.
Shifting gears: Diversification, intensification, and effort increases in small-scale fisheries (1950-2010)

S1 Table. Summary information about 23 fishing communities in the central Danajon Bank, Philippines including population sizes, sample sizes, and the presence of fishers' organizations and MPAs.

Province	Municipality	Location	Village	Village Population (2010)	No. Fishers in Village (2010)	No. Fishers Interviewed (2010)	Fisher Organization (Presence)	MPA (Year Started)
Bohol	Buena Vista	Caye	Cabul-an West	1884	349	28	1	2000
Bohol	Buena Vista	Mainland	Asinan	757	71	5	1	2000
Bohol	Buena Vista	Mainland	Cruz	745	38	9	1	none
Bohol	Getafe	Caye	Nasingin	1441	335	25	1	2002
Bohol	Getafe	Caye	Pandanon	1833	320	21	1	2002
Bohol	Getafe	Ter. Island	Jandayan Norte[a]	694	23	11	1	2002
Bohol	Getafe	Ter. Island	Mahanay	448	21	2	1	2003
Bohol	Getafe	Mainland	Carlos P. Garcia	725	21	1	0	none
Bohol	Getafe	Mainland	Corte Baud	622	82	4	1	2002
Bohol	Getafe	Mainland	Poblacion	2210	36	3	1	none
Bohol	Getafe	Mainland	San Jose	1441	50	3	0	none
Bohol	Getafe	Mainland	Tugas	817	58	4	1	2002
Bohol	Getafe	Mainland	Tulang	1594	75	6	1	2003
Bohol	Inabanga	Mainland	Lawis	2629	447	31	1	2000
Bohol	Inabanga	Mainland	Ondol	1098	131	9	1	2000
Bohol	Talibon	Caye	Calituban	4527	552	37	1	2000
Bohol	Talibon	Ter. Island	Mahanay	2012	113	8	1	unclear
Bohol	Talibon	Mainland	Bagacay	3106	213	16	0	none
Bohol	Talibon	Mainland	San Francisco	5870	408	25	1	1996
Cebu	Lapu Lapu	Caye	Caubian	2114	429	30	1	2007
Cebu	Lapu Lapu	Ter. Island	Baring	2934	530	37	0	2003
Cebu	Lapu Lapu	Ter. Island	Talima	4945	829	58	0	2003
Cebu	Lapu Lapu	Ter. Island	Tungasan	1754	242	18	0	none

[a]Number estimated from Getafe Coastal Resource Management Plan (2001 - 2005)
Data Sources: Village Population = Census data collected by Village Health Centers; No. of Fishers in Village = Based on census data and our interviews with health workers and other relevant village officials, MPA = expert interviews, government websites and Alcala et al. (2008)
Note: Ter. = Terrestrial
