# Supplementary material for: Shifting gears: Diversification, intensification, and effort increases in small-scale fisheries (1950-2010)
Source: PLoS One. 2018 Mar 14;13(3):e0190232. doi: 10.1371/journal.pone.0190232 (PMC5851533; doi:10.1371/journal.pone.0190232)
Supplement: S2 Table — Full models tested the effects of year and governance period, and their interaction on total fishing effort by each category of fishing gear. Models considered changes in the use of eight general classes of fishing gears and four pairs of intensive/non-intensive gear categories. (PDF) [file pone.0190232.s002.pdf]

Jennifer C. Selgrath, Sarah E. Gergel, and Amanda C. J. Vincent.
Shifting gears: Growing effort, impacts, and diversity in small-scale fisheries (1950-2010)

S2 Table. Summary of GLS model statistics for changes in the total fishing effort (fishing days per year by fishers in participating villages) allocated to gears in the central Danajon Bank, Philippines (1960-2010). Full models tested the effects of year and governance period, and their interaction on total fishing effort by each category of fishing gear. Models considered changes in the use of eight general classes of fishing gears and four pairs of intensive/non-intensive gear categories.

Model	Variable	Coefficient Effect	F-value	p-value
**Four common fishing gears**				
Net				
	Intercept	-	5173.26	< 0.001
	Year	+	386.83	< 0.001
Dive				
	Intercept	-	38.54	< 0.001
	Year	+	47.45	< 0.001
Hook				
	Intercept	-	977.98	< 0.001
	Year	+	4.20	0.05
Trap				
	Intercept	+	2520.37	< 0.001
	Year	-	1.77	0.19
	Governance era		9.18	< 0.001
	Governance era (Productivity)	+		
	Governance era (Decentralized)	-		
	Governance era (Co-management)	-		
	Year:Governance era		9.69	< 0.001
	Year:Governance era (Productivity)	-		
	Year:Governance era (Decentralized)	+		
	Year:Governance era (Co-management)	+		

Model	Variable	Coefficient Effect	F-value	p-value
**Four uncommon fishing gears**				
Blast				
	Intercept	+	1060.69	< 0.001
	Year	-	0.18	0.67
	Governance era		5.68	< 0.01
	Governance era (Productivity)	-		
	Governance era (Decentralized)	-		
	Governance era (Co-management)	-		
	Year:Governance era		22.05	< 0.001
	Year:Governance era (Productivity)	+		
	Year:Governance era (Decentralized)	+		
	Year:Governance era (Co-management)	+		
Poison				
	Intercept	+	481.49	< 0.001
Gleaning				
	Intercept	-	229.10	< 0.001
	Year	+	2.78	0.10
	Governance era		2.23	0.10
	Governance era (Productivity)	+		
	Governance era (Decentralized)	+		
	Governance era (Co-management)	-		
	Year:Governance era		3.65	0.02
	Year:Governance era (Productivity)	-		
	Year:Governance era (Decentralized)	-		
Corral				
	Intercept	-	977.98	< 0.001
	Year	+	4.20	0.05
	Year:Governance era (Co-management)	+		

Model	Variable	Coefficient Effect	F-value	p-value
**Intensive fishing gears**				
Destructive				
	Intercept	-	59.43	< 0.001
	Year	+	13.63	< 0.001
Active				
	Intercept	-	437.89	< 0.001
	Year	+	79.33	< 0.001
Non-selective				
	Intercept	-	90.93	< 0.001
	Year	+	52.75	< 0.001
Illegal				
	Intercept	+	64.14	< 0.001
	Year	-	44.15	< 0.001
	Governance era		28.32	< 0.001
	Governance era (Productivity)	-		
	Governance era (Decentralized)	-		
	Governance era (Co-management)	-		
	Year:Governance era		2.23	0.10
	Year:Governance era (Productivity)	+		
	Year:Governance era (Decentralized)	+		
	Year:Governance era (Co-management)	+		

Model	Variable	Coefficient Effect	F-value	p-value
**Non-intensive fishing gears**				
Non-destructive				
	Intercept	-	42.72	< 0.001
	Year	+	32.04	< 0.001
Passive				
	Intercept	-	144.39	< 0.001
	Year	+	9.70	< 0.01
	Governance era			
	Governance era (Productivity)	+	0.10	0.96
	Governance era (Decentralized)	+	2.50	0.07
	Governance era (Co-management)	-		
	Year:Governance era			
	Year:Governance era (Productivity)	-		
	Year:Governance era (Decentralized)	-		
	Year:Governance era (Co-management)	-		
Selective				
	Intercept	-	159.37	< 0.001
	Year	+	4.71	0.03
Legal				
	Intercept	-	831.04	< 0.001
	Year	+	55.07	< 0.001
	Governance era		19.25	< 0.001
	Governance era (Productivity)	+		
	Governance era (Decentralized)	-		
	Governance era (Co-management)	-		
	Year:Governance era		1.81	0.16
	Year:Governance era (Productivity)	-		
	Year:Governance era (Decentralized)	+		
	Year:Governance era (Co-management)	+		
