# Supplementary material for: Shifting gears: Diversification, intensification, and effort increases in small-scale fisheries (1950-2010)
Source: PLoS One. 2018 Mar 14;13(3):e0190232. doi: 10.1371/journal.pone.0190232 (PMC5851533; doi:10.1371/journal.pone.0190232)
Supplement: S3 Table — Full models tested the effects of year and governance period, and their interaction on relative fishing effort by each category of fishing gear. Models considered changes in the use of eight general classes of fishing gears and four pairs of intensive/non-intensive gear categories. (PDF) [file pone.0190232.s003.pdf]

**Jennifer C. Selgrath, Sarah E. Gergel, and Amanda C. J. Vincent.**
**Shifting gears: Diversification, intensification, and effort increases in small-scale fisheries (1950-2010)**

**S3 Table. Summary of GLS model statistics for changes in the relative fishing effort (percentage of fishing days per year) allocated to gears in the central Danajon Bank, Philippines (1960-2010).** Full models tested the effects of year and governance period, and their interaction on relative fishing effort by each category of fishing gear. Models considered changes in the use of eight general classes of fishing gears and four pairs of intensive/non-intensive gear categories.

| Model | Variable | Coefficient Effect | F-value | p-value |
|---|---|---|---|---|
| **Four common fishing gears** | | | | |
| *Hook* | | | | |
| | Intercept | + | 2343.57 | < 0.001 |
| | Year | - | 58.74 | < 0.001 |
| *Net* | | | | |
| | Intercept | + | 5068.87 | < 0.001 |
| | Year | - | 4.28 | 0.04 |
| | Governance era | | 2.80 | 0.05 |
| | Governance era (Productivity) | - | | |
| | Governance era (Decentralized) | - | | |
| | Governance era (Co-management) | - | | |
| | Year:Governance era | | 6.90 | < 0.001 |
| | Year:Governance era (Productivity) | + | | |
| | Year:Governance era (Decentralized) | + | | |
| | Year:Governance era (Co-management) | + | | |
| *Poison* | | | | |
| | Intercept | + | 744.30 | < 0.001 |
| | Governance era | | 4.50 | < 0.01 |
| | Governance era (Productivity) | + | | |
| | Governance era (Decentralized) | + | | |
| | Governance era (Co-management) | + | | |
| *Trap* | | | | |
| | Intercept | + | 1379.63 | < 0.001 |
| | Year | - | 23.81 | < 0.001 |
| | Governance era | | 4.00 | 0.01 |
| | Governance era (Productivity) | + | | |
| | Governance era (Decentralized) | - | | |
| | Governance era (Co-management) | - | | |
| | Year:Governance era | | 5.21 | < 0.01 |
| | Year:Governance era (Productivity) | - | | |
| | Year:Governance era (Decentralized) | + | | |
| | Year:Governance era (Co-management) | + | | |

| Model | Variable | Coefficient Effect | F-value | p-value |
|---|---|---|---|---|
| **Four uncommon fishing gears** | | | | |
| *Blast* | | | | |
| | Intercept | + | 1163.85 | < 0.001 |
| | Year | - | 27.65 | < 0.001 |
| | Governance era | | 7.23 | < 0.001 |
| | Governance era (Productivity) | - | | |
| | Governance era (Decentralized) | - | | |
| | Governance era (Co-management) | - | | |
| | Year:Governance era | | 23.63 | < 0.001 |
| | Year:Governance era (Productivity) | + | | |
| | Year:Governance era (Decentralized) | + | | |
| | Year:Governance era (Co-management) | + | | |
| *Corral* | | | | |
| | Intercept | + | 2343.57 | < 0.001 |
| | Year | - | 58.74 | < 0.001 |
| *Dive* | | | | |
| | Intercept | - | 635.79 | < 0.001 |
| | Year | + | 27.67 | < 0.001 |
| *Gleaning* | | | | |
| | Intercept | + | 123.90 | < 0.001 |

| Model | Variable | Coefficient Effect | F-value | p-value |
|---|---|---|---|---|
| **Intensive fishing gears** | | | | |
| *Destructive* | | | | |
| | Intercept | + | 218.92 | < 0.001 |
| *Active* | | | | |
| | Intercept | - | 1113.37 | < 0.001 |
| | Year | + | 4.25 | 0.04 |
| *Non-selective* | | | | |
| | Intercept | - | 2475.90 | < 0.001 |
| | Year | + | 17.76 | < 0.001 |
| *Illegal* | | | | |
| | Intercept | + | 70.78 | < 0.001 |
| | Year | - | 136.68 | < 0.001 |
| | Governance era | | 55.97 | < 0.001 |
| | Governance era (Productivity) | - | | |
| | Governance era (Decentralized) | - | | |
| | Governance era (Co-management) | - | | |
| | Year:Governance era | | 1.30 | 0.29 |
| | Year:Governance era (Productivity) | + | | |
| | Year:Governance era (Decentralized) | + | | |
| | Year:Governance era (Co-management) | + | | |

| Model | Variable | Coefficient Effect | F-value | p-value |
|---|---|---|---|---|
| **Non-intensive fishing gears** | | | | |
| *Non-destructive* | | | | |
| | Intercept | + | 3382.55 | < 0.001 |
| | Year | - | 0.30 | 0.58 |
| *Passive* | | | | |
| | Intercept | + | 1466.29 | < 0.001 |
| | Year | - | 4.25 | 0.04 |
| *Selective* | | | | |
| | Intercept | + | 805.05 | < 0.001 |
| | Year | - | 17.76 | < 0.001 |
| *Legal* | | | | |
| | Intercept | - | 124894.29 | < 0.001 |
| | Year | + | 136.68 | < 0.001 |
| | Governance era | | 55.97 | < 0.001 |
| | Governance era (Productivity) | + | | |
| | Governance era (Decentralized) | + | | |
| | Governance era (Co-management) | + | | |
| | Year:Governance era | | 1.30 | 0.29 |
| | Year:Governance era (Productivity) | - | | |
| | Year:Governance era (Decentralized) | - | | |
| | Year:Governance era (Co-management) | - | | |
