# Supplementary material for: Shifting gears: Diversification, intensification, and effort increases in small-scale fisheries (1950-2010)
Source: PLoS One. 2018 Mar 14;13(3):e0190232. doi: 10.1371/journal.pone.0190232 (PMC5851533; doi:10.1371/journal.pone.0190232)
Supplement: S4 Table — Full models tested the effects of year and governance period, and their interaction on the percentage of fishers by each category of fishing gear. Models considered changes in the use of eight general classes of fishing gears and four pairs of intensive/non-intensive gear categories. (PDF) [file pone.0190232.s004.pdf]

Jennifer C. Selgrath, Sarah E. Gergel, and Amanda C. J. Vincent.
Shifting gears: Diversification, intensification, and effort increases in small-scale fisheries (1950-2010)

S4 Table. Summary of GLS model statistics for changes in the proportion of fishers using various fishing gears in a year in the central Danajon Bank, Philippines (1960-2010). Full models tested the effects of year and governance period, and their interaction on the percentage of fishers by each category of fishing gear. Models considered changes in the use of eight general classes of fishing gears and four pairs of intensive/non-intensive gear categories.

Model	Variable	Coefficient Effect	F-value	p-value
**Four common fishing gears**				
Net				
	Intercept	-	1999.79	< 0.001
	Year	+	12.10	< 0.01
Dive				
	Intercept	-	720.71	< 0.001
	Year	+	15.19	< 0.001
	Governance era		0.48	0.70
	Governance era (Productivity)	+		
	Governance era (Decentralized)	+		
	Governance era (Co-management)	+		
	Year:Governance era		1.87	0.15
	Year:Governance era (Productivity)	-		
	Year:Governance era (Decentralized)	-		
	Year:Governance era (Co-management)	-		
Hook				
	Intercept	-	6184.29	< 0.001
	Year	+	27.01	< 0.001
	Governance era		2.04	0.12
	Governance era (Productivity)	+		
	Governance era (Decentralized)	+		
	Governance era (Co-management)	+		
	Year:Governance era		8.06	< 0.001
	Year:Governance era (Productivity)	-		
	Year:Governance era (Decentralized)	-		
	Year:Governance era (Co-management)	-		
Trap				
	Intercept	+	715.12	< 0.001

Model	Variable	Coefficient Effect	F-value	p-value
**Four uncommon fishing gears**				
Blast				
	Intercept	+	300.77	< 0.001
	Year	-	2.96	0.09
	Governance era		0.07	0.98
	Governance era (Productivity)	-		
	Governance era (Decentralized)	-		
	Governance era (Co-management)	-		
	Year:Governance era		6.63	< 0.001
	Year:Governance era (Productivity)	+		
	Year:Governance era (Decentralized)	+		
	Year:Governance era (Co-management)	+		
Poison				
	Intercept	-	102.95	< 0.001
	Year	+	4.20	0.045912
Gleaning				
	Intercept	+	306.90	< 0.001
Corral				
	Intercept	-	6184.29	< 0.001
	Year	+	27.01	< 0.001
	Governance era		2.04	0.12
	Governance era (Productivity)	+		
	Governance era (Decentralized)	+		
	Governance era (Co-management)	+		
	Year:Governance era		8.06	< 0.001
	Year:Governance era (Productivity)	-		
	Year:Governance era (Decentralized)	-		
	Year:Governance era (Co-management)	-		

Model	Variable	Coefficient Effect	F-value	p-value
**Intensive fishing gears**				
Destructive				
	Intercept	+	469.06	< 0.001
Active				
	Intercept	+	2057.85	< 0.001
	Year	-	3.65	0.06
	Governance era		0.33	0.81
	Governance era (Productivity)	-		
	Governance era (Decentralized)	-		
	Governance era (Co-management)	-		
	Year:Governance era		2.56	0.07
	Year:Governance era (Productivity)	+		
	Year:Governance era (Decentralized)	+		
	Year:Governance era (Co-management)	+		
Non-selective				
	Intercept	-	2594.48	< 0.001
	Year	+	22.78	< 0.001
Illegal				
	Intercept	+	492.85	< 0.001
	Year	-	378.33	< 0.001
	Governance era		148.44	< 0.001
	Governance era (Productivity)	-		
	Governance era (Decentralized)	-		
	Governance era (Co-management)	-		
	Year:Governance era		7.49	< 0.001
	Year:Governance era (Productivity)	+		
	Year:Governance era (Decentralized)	+		
	Year:Governance era (Co-management)	+		

Model	Variable	Coefficient Effect	F-value	p-value
**Non-intensive fishing gears**				
Non-destructive				
	Intercept	-	38997.43	< 0.001
	Year	+	0.17	0.685846
	Governance era		5.90	< 0.01
	Governance era (Productivity)	+		
	Governance era (Decentralized)	+		
	Governance era (Co-management)	-		
	Year:Governance era		4.45	< 0.01
	Year:Governance era (Productivity)	-		
	Year:Governance era (Decentralized)	-		
	Year:Governance era (Co-management)	+		
Passive				
	Intercept	+	1833.84	< 0.001
	Year	-	0.07	0.785763
	Governance era		1.29	0.291541
	Governance era (Productivity)	+		
	Governance era (Decentralized)	+		
	Governance era (Co-management)	-		
	Year:Governance era		3.27	0.030295
	Year:Governance era (Productivity)	-		
	Year:Governance era (Decentralized)	-		
	Year:Governance era (Co-management)	+		
Selective				
	Intercept	+	1304.21	< 0.001
Legal				
	Intercept	-	753180619.02	< 0.001
	Year	+	1386.13	< 0.001
	Governance era		36.73	< 0.001
	Governance era (Productivity)	+		
	Governance era (Decentralized)	+		
	Governance era (Co-management)	+		
	Year:Governance era		5.54	< 0.01
	Year:Governance era (Productivity)	-		
	Year:Governance era (Decentralized)	-		
	Year:Governance era (Co-management)	-		
